# Non-compliance with a postmastectomy radiotherapy guideline: Decision tree and cause analysis

**DOI:** 10.1186/1472-6947-8-41

**Published:** 2008-09-21

**Authors:** Amir R Razavi, Hans Gill, Hans Åhlfeldt, Nosrat Shahsavar

**Affiliations:** 1 Department of Biomedical Engineering, Division of Medical Informatics, Linköping University, Sweden; 2 Regional Oncology Centre, University Hospital, Linköping, Sweden

## Abstract

**Background:**

The guideline for postmastectomy radiotherapy (PMRT), which is prescribed to reduce recurrence of breast cancer in the chest wall and improve overall survival, is not always followed. Identifying and extracting important patterns of non-compliance are crucial in maintaining the quality of care in Oncology.

**Methods:**

Analysis of 759 patients with malignant breast cancer using decision tree induction (DTI) found patterns of non-compliance with the guideline. The PMRT guideline was used to separate cases according to the recommendation to receive or not receive PMRT. The two groups of patients were analyzed separately. Resulting patterns were transformed into rules that were then compared with the reasons that were extracted by manual inspection of records for the non-compliant cases.

**Results:**

Analyzing patients in the group who should receive PMRT according to the guideline did not result in a robust decision tree. However, classification of the other group, patients who should not receive PMRT treatment according to the guideline, resulted in a tree with nine leaves and three of them were representing non-compliance with the guideline. In a comparison between rules resulting from these three non-compliant patterns and manual inspection of patient records, the following was found:

In the decision tree, presence of perigland growth is the most important variable followed by number of malignantly invaded lymph nodes and level of Progesterone receptor. DNA index, age, size of the tumor and level of Estrogen receptor are also involved but with less importance. From manual inspection of the cases, the most frequent pattern for non-compliance is age above the threshold followed by near cut-off values for risk factors and unknown reasons.

**Conclusion:**

Comparison of patterns of non-compliance acquired from data mining and manual inspection of patient records demonstrates that not all of the non-compliances are repetitive or important. There are some overlaps between important variables acquired from manual inspection of patient records and data mining but they are not identical. Data mining can highlight non-compliance patterns valuable for guideline authors and for medical audit. Improving guidelines by using feedback from data mining can improve the quality of care in oncology.

## Background

Recurrence of breast cancer is a major concern for patients and health systems around the world. Most of them occur in the first five years after initial treatment and can be in the form of a local or a distant recurrence in the body [1]. Many patients with breast cancer are treated with breast conserving surgery rather than mastectomy. However, a considerable percentage of women still require or choose the surgical procedure of mastectomy, which is complete surgical removal of one or both breasts [2]. To reduce recurrence of cancer in the chest wall and improve overall survival, radiotherapy after mastectomy, or postmastectomy radiotherapy (PMRT) of the chest wall and the regional lymph nodes, is advised [3,4].

Clinical benefits related to PMRT depend on the treatment technique and the risk of breast cancer recurrence. However, there is a trade-off between the benefits and the adverse effects of this treatment. Therefore, a guideline based on evidence obtained from different studies is used for prescribing PMRT to patients who have undergone mastectomy as their primary surgical treatment [5,6]. If clinical guidelines are implemented successfully, the results will generally be reduced costs and length of stay in the hospital, minimized variations in medical practice, and increased quality of care and patient satisfaction [7].

However, not all patients are treated according to guidelines. A number of reasons for this have been studied, such as physicians' disagreement with guidelines [8]. Some of the barriers are associated with patient-related obstacles [9]. In oncology, this can be due to patient characteristics including tumor specifications such as size, location and age. Patients may also have co-morbidities or may not accept the suggested treatment.

Among different causes of inconsistencies between guidelines and the actual treatment, some reasons may be retrieved from cancer quality registries. These registries contain individual-based data on diagnoses, treatments and outcomes and they play an important role in the control and improvement of health care quality [10].

A method for finding disagreements is to apply the PMRT guideline to each case and compare the result with the actual treatment received by the patient. Manual inspection is mainly done retrospectively by medical experts and is time consuming. The deployment of automatic or semi-automatic methods for systematic follow-up of compliance based on the availability of proper data sources would therefore be a great improvement in the field [11].

Clinical guidelines can be transformed into computer-processable rules and integrated with interactive decision support systems for providing feedback on decisions associated with an individual patient [11]. However, a main criterion is that necessary variables regarding the patient's condition and treatment should be available in the data source.

Data mining as a method for discovering meaningful new patterns and trends [12] has in our case been used for highlighting non-compliance with a guideline. With this method, data can be analyzed in order to find repetitive patterns and disagreements with a guideline. If proven successful, instead of manually evaluating each case with a guideline, a whole set of patients can be investigated automatically. Several studies have used data mining methods to find patterns in non-compliance with clinical guidelines for diseases such as hypertension [13,14].

Variables of importance when prescribing PMRT, such as age, tumor size and number of involved lymph nodes, are present in the breast cancer registry. Therefore, analyzing this registry using guideline rules can identify cases that were not treated according to the guideline. It is also possible to partition patients according to the recommendations from guideline to patients who should or should not receive the treatment. After identifying patterns for non-compliance with the guideline, they should be verified via manual inspection of patient medical records.

Decision tree models can visualize classification based on a set of variables. The models can be used as a basis for discussions about reasons for non-compliance. Furthermore, if some patterns are shown to be important in causing non-compliance with the guideline, they should be further investigated and reported to guideline authors.

The objective of this study was to identify patterns of non-compliance with the PMRT guideline by studying a dataset from a breast cancer registry. Patterns identified by data mining were compared to reasons identified by manual inspection of patient records.

## Methods

By means of a locally adopted version of PMRT guideline rules, cases were divided into two groups of patients, those who should respectively should not receive PMRT according to the guideline.

These two groups were analyzed with decision tree induction (DTI) to find noteworthy patterns of inappropriate PMRT prescription. Medical records for each of the patients that were not treated according to the guideline were reviewed and the reasons for non-compliance were extracted and categorized. Reasons acquired with these two approaches, data mining and manual inspection, were then compared.

### Data Source and Variables

Data were collected from 759 female patients with the diagnosis of malignant breast cancer and primary surgical treatment with mastectomy. Patients were admitted to Linköping University Hospital, and the earliest patient was diagnosed on 1 January 1990 and the last one on 29 December 2000. During this period, the same PMRT guideline was used for treating patients.

Variables from the tumor marker registry were linked and matched with the main dataset. The tumor marker registry contains information about tumor markers for breast cancer. After matching and retrieval of data, we could gather data for twelve variables. These variables were age of the patient at the time of diagnosis, number of involved lymph nodes, tumor size, presence of multiple tumor, location of the tumor, presence of perigland growth, receptors for estrogen and progesterone, S-phase fraction, DNA index, DNA ploidy and if the patient has received PMRT treatment.

Thereafter, missing values for continuous variables in the original dataset were handled using the expectation maximization (EM) method [15]. Three variables, namely perigland growth, presence of multiple tumor and location of the tumor, were binary variables. Among these variables, only presence of multiple tumor had 5% missing values that were omitted from the study which resulted in 759 cases.

The analysis of patient records was approved by the Linköping University ethics committee. No information or clinical photographs relating to individual patient were stated in this study.

### The Guideline and Non-compliant Cases

In this study, adherence to the guideline for postmastectomy radiotherapy was studied [6]. The original national guideline was adopted at Linköping University Hospital in order to conform to local experience [5]. A major modification was to suggest the treatment to patients with involvement of one or more than one lymph node.

With knowledge of the actual treatment for each patient, a table was constructed showing the recommendations from the PMRT guideline and whether or not the patient received this treatment. The cases that were not treated in accordance with the guideline were identified and extracted for further analysis. In order to process a comparison between the dataset and the guideline, the textual guideline was manually converted to computer-processable format. Then these rules were applied to the dataset as SQL queries to find cases that were not treated according to the PMRT guideline.

In order to perform a comparison between the dataset and the guideline, the textual guideline was manually converted to computer-processable format. First, the variables referred to in the guideline were identified and mapped to the dataset, whereafter SQL queries were constructed corresponding to the logic of the guideline. Then these SQL queries were applied to the database to identify cases that were not treated according to the PMRT guideline. The mapping procedure was performed by two of the authors (ARR, NS) and checked by an experienced oncologist. Because of the limited size of the PMRT guideline, the whole process could be done manually. For more complex and longer guidelines, guideline modelling methodologies such as GEM [16], PROforma [17] or GLIF [18] should have been used.

As a quality control procedure, a biomedical analyst with long experience in working with the breast cancer registry used the ID numbers for these cases to find their medical records. After reading the records, reasons for not following the guideline were extracted, categorized and documented in a study protocol under the supervision of an experienced oncologist. In some of the cases, such as when the patient rejected the treatment, explicit reasons for not following the guideline were documented in the medical records. If the reason was not mentioned, such as cases with near cut-off level values for risk factors, the biomedical analyst discussed the findings with the experienced oncologist for pointing out reasons for non-compliance.

### Decision Tree Induction

Two groups of patients separated according to the PMRT guideline were analyzed. One group consisted of patients that according to the guideline should receive the treatment (329 cases) and the other group included patients who should not receive PMRT treatment (430 cases).

These groups were analyzed by data mining to group non-compliant cases according to the set of variables. Decision tree induction (DTI) was used to classify these cases. DTI uses information gain as a heuristic for selecting the variable that will best separate the cases into each outcome. In a decision tree, each internal node denotes a test on a variable, and each branch stands for an outcome of the test. Leaf nodes represent an outcome, and the uppermost node in a tree is the root node. The tree can be easily transformed to rules and integrated into computer applications. The ability to create an understandable representation of a classified dataset make decision trees one of the most frequently used data mining techniques [19]. For validating the result, 10-fold stratified cross validation was used. In this method, the data were randomly divided into ten groups with equal proportions of PMRT. Nine groups were used as the training set, and one was left for testing. This process was repeated ten times and at the end the error rates were calculated as the average of ten iterations of training and testing.

## Results

**Figure 1:**
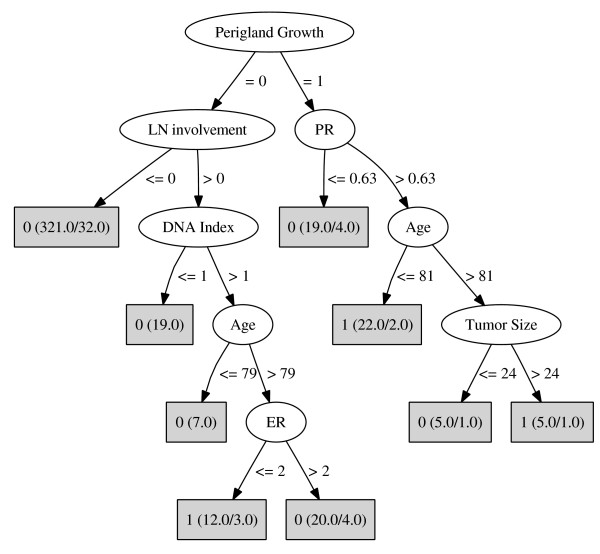
**Decision tree resulting from analyzing patients who should have not received PMRT treatment according to the guideline**. In the leaves (gray boxes), there are two numbers in parentheses. The first number shows the number of cases who reached this leaf and the second shows the number of cases for whom the leaf class was not predicted to happen. The number outside the parentheses indicates the class for cases that reach this leaf. 1 means non-compliant cases and 0 means compliant cases with PMRT guideline.

125 cases (Table 1) were found to have been treated in contrast to the PMRT guideline. Analysis of cases that should receive PMRT according to the guideline did not result in any decision tree that could discriminate between compliant and non-compliant cases. On the other hand, analyzing the other group consisting of patients who should not receive PMRT treatment according to the guideline resulted in a decision tree with the size of seventeen nodes and nine leaves. This decision tree is shown in Figure 1. Three of the leaves show non-compliant cases, i.e. cases that received PMRT treatment in contrast to the guideline. In Table 2, pathways from the root node to these three non-compliance leaves are shown as rules.

**Table 1:** Contingency table for PMRT and decision according to the PMRT guideline.

		**Guideline PMRT**	
		
		**-**	**+**
**Dataset PMRT**	**-**	356	51
	**+**	74	278

**Table 2:** Left-hand side for the rules acquired from data mining in cases that are not recommended to receive PMRT but in contrast to the guideline have received PMRT.


No. 1	There are no perigland growth AND some lymph nodes are involved by malignant cells AND DNA index is more than 1 AND patient is older than 79 years old AND Estrogen receptor level is less than or equal to 2 fmol/mg

No. 2	Perigland growth is present AND Progesterone level is more than 0.63 fmol/mg AND patient is younger than 81 years old

No. 3	Perigland growth is present AND Progesterone level is more than 0.63 fmol/mg AND patient is older than 81 years old AND tumor is larger than 24

Regarding manual inspection, reasons for not following PMRT guideline were extracted from the hospital information system and the patient records and illustrated in Table 3 and Table 4. Some of the cases had a combination of reasons and all of them are presented in the tables. Table 5 shows the comparison between reasons for prescribing PMRT in contrast to the guideline and DTI.

**Table 3:** Reasons for prescribing PMRT, in contrast to the guideline.

	**Reasons**	**Number**
Physician-related		**4**

Patient-related	Age above the threshold	**40**
	Near cut-off level values for RFs	**13**
	Advanced disease	**4**

Registry-related	Reporting error	**11**
	RT after local relapse	**4**

GL was followed		**1**

Unknown reason		**4**

RF: Risk factors, RT: Radiotherapy, GL: Guideline

**Table 4:** Reasons for not prescribing PMRT, in contrast to the guideline.

	**Reasons**	**Number**
Physician-related		**5**

Patient-related	Co-morbidities	**14**
	Rejected the treatment	**3**
	Generalized disease	**9**
	Early recurrence	**5**
	Preoperative RT	**3**

Registry-related		**1**

GL was followed		**1**

Unknown reason		**13**

RT: Radiotherapy, GL: Guideline

**Table 5:** Results from data mining and manual inspection of records for cases who received PMRT treatment in contrast to the guideline.

	**Reasons**	**Number**		
		
		**Rule 1**	**Rule 2**	**Rule 3**
Patient-related	Age above the threshold	**8**	**20**	**4**
	Near cut-off level values for RFs	**-**	**2**	**-**
Unknown reason		**1**	**-**	**-**

RFs: Risk factors

## Discussion

In order to identify noteworthy patterns of inappropriate PMRT treatment, data mining was used to analyze groups of patients already partitioned according to the guideline. Comparison between the result from data mining and manual inspection of non-compliant cases showed that only in one of the groups, discrimination between compliant/non-compliant cases is meaningful. A detailed discussion follows concerning comparison between results from data mining and the manual inspection of cases.

### Group One

This group consisted of patients who should not receive PMRT treatment according to the guideline. Generally, these patients do not benefit from PMRT because the disease is in earlier stages [6] or they are not good candidates for PMRT for example due to comorbidities or being too elderly. Manual inspection of cases in this group using the guideline shows that the most common characteristic among non-compliant cases was being older than 75 years, or having near cut-off level values for risk factors. In some cases, the documentation in the patient record did not reveal any specific reason for non-compliance (Table 5).

Age is directly related to the presence of comorbidities. Older patients tend to have more comorbidities, and in that case they are not good candidates for PMRT treatment.

The most important variable identified by DTI was the presence of perigland growth. This means that for discriminating between compliant/non-compliant, this is the most important variable. More variables characterizing non-compliant cases identified by DTI are number of lymph nodes that are invaded by malignant cells, Progesterone receptor level, DNA index, age, tumor size and Estrogen receptor level. If variables are ranked according to their importance, two variables from the guideline, lymph node involvement status and age are placed in the middle of the list. This can show that physicians consider more risk factors for prescribing PMRT. Simply being older than 75 years is apparently not a sufficient reason for not to prescribe PMRT. It is however more common for older patients to have other diseases and complications, which makes them less favourable candidates for receiving PMRT.

This indicates that the above-mentioned variables may be further investigated and reported to guideline authors for consideration.

A better evaluation of non-compliance may be done by applying fuzzy set theory (FST). If two patients who received PMRT are 76 and 85 years old, both are older than 75 years but the degree of non-compliance to the guideline is different. Fuzzification of variable cut-offs can give a more realistic estimation of non-compliance with the guideline.

### Group Two

This group consisted of patients who should receive PMRT treatment according to the guideline. PMRT guideline is based on evidence obtained from different studies and it is shown that certain patients benefit from this treatment in the form of reduced recurrence of the disease and also improving survival of the patients [20,21]. Therefore, non-compliance with the PMRT guideline can negatively affect recurrence of the disease and survival of these patients and repeated and important patterns should be acted upon immediately.

Analyzing this group of patients with data mining did not result in a meaningful decision tree. Thus it was not possible to discriminate patients according to compliance with the guideline. If there was a pattern for non-compliance with the guideline, most probably DTI should have identified it. Absence of such patterns indicate that there is no repetitive or noteworthy pattern in not following PMRT guideline for this group of patients, or that the cancer registry does not contain relevant variables for this group. Reasons for refraining from PMRT are probably more patient-related and unlikely to be present in the cancer registry.

Manual inspection of non-compliant cases in this group (Table 4) reveals that the main reason for not prescribing PMRT was the presence of co-morbidities (presence of one or more diseases in addition to breast cancer) and a generalized disease. These two variables are not usually reported in breast cancer registries. When physicians deduce that the general condition of a patient is not good and the disease is advanced, PMRT is not prescribed.

### Cancer registries and non-compliances

Analyzing a regional breast cancer registry using PMRT guideline rules reveals cases that are not treated according to the guideline. This is a new way of using cancer registries, which are traditionally used for calculating survival of cancer patients. In this case, the most important advantage of using guideline rules is to save time in finding these cases. In controlled studies, it is possible to manually inspect each patient's file. In routine clinical care, it is not feasible to expect a medical expert to do this in order to see whether a patient was treated according to one or a set of guidelines. Therefore, any data source and method that can contribute to systematic follow-up of guideline compliance is beneficial. A combination of guideline rules and DTI for analyzing data in a cancer registry is an option.

There are reporting errors in 12 cases as shown in Table 3 and Table 4. Therefore, before using a breast cancer registry for finding cases that were not treated according to the PMRT guideline, a margin of registration error should be considered. Discovering reporting errors may also improve the data quality for further analyses and should result in better quality control mechanisms. Rather low quality of the dataset, i.e. due to reporting errors could compromise the analysis results. In order to investigate this, the dataset has been re-analysed without the reporting error cases. However, no important difference was noticed between the results, showing the robustness of data mining to find important patterns with respect to reporting errors.

After identifying cases that were not treated according to the guideline, the cases can be further investigated by manual inspection of patient records. Furthermore, to take advantage of new intelligent analytical methods, it is beneficial to use data mining methods to extract a model for viewing important non-compliance patterns. Discovered reasons for non-compliance will of course be limited to variables that are stored in the registries. It is also beneficial to investigate patients belonging to these important patterns separately, and in detail, by reviewing their records. Missing values in PMRT is another factor that affects identification of non-compliance patterns.

In some patients (17 cases), no obvious reason for non-compliance with the guideline could be found. This is an interesting finding, indicating that efforts should be made to achieve a systematic and structured way of documenting clinical decision making in patient records.

Some studies have been conducted in which data mining techniques were applied in order to find cases of non-compliance with a specific guideline. Svatek et al. [14] examined the automatic detection of potential explanations of non-compliance by implementing association-mining in the domain of hypertension management. They proposed that frequent non-compliance patterns could be submitted to medical experts for interpretation and they examined their methodology with 48 patients. Our approach, on the other hand, focused on comparisons between patterns discovered by DTI from a regional cancer registry and reasons acquired by manual inspection of patient records in the domain of oncology.

Marcos et al. [22] studied compliance with guidelines in the domain of neonatal jaundice. Experts' solutions for a set of cases were manually compared with those provided by the formalized guideline. They identified some non-compliance patterns as local deviations, potential gaps in the guidelines, and some artefacts of imperfect guideline formalization. In our approach, physicians' decisions were recorded as historical data in the registry and their routine practices were studied. Asking their opinion about individual patients may have added some bias, because they would be prepared to give the best possible answer about cases and to propose management that was similar to the guideline. Our focus was to see if breast cancer registries could help to identify cases that were non-compliant with the PMRT guideline and compare patterns resulting from mining these historical data with reasons acquired through manual inspection of patient records.

### Future Work

Planning is underway to study the survival of patients and to compare this between two groups, those who received the right treatment according to the PMRT guideline and those who did not. Testing the proposed methodology with other data sources is another continuation of this work.

## Conclusion

Analyzing a regional breast cancer registry using guideline rules for PMRT enables automatic detection of cases that were not treated according to the guideline. Data mining can reveal patterns of non-compliance with a guideline provided that the variables referred to in the guideline are recorded in the registry.

## Abbreviations

DTI: Decision Tree Induction; PMRT: Postmastectomy Radiotherapy; EM: Expectation Maximization; FST: Fuzzy Set Theory.

## Competing interests

The authors declare that they have no competing interests.

## Authors' contributions

ARR, HÅ, NS contributed to the conception of the study. ARR is the main author and designed the study, prepared and analyzed the data and interpreted the result. HG contributed to preparation and analysis of the data, interpretation of the result and revision of the article. HÅ and NS contributed to the design of the study and revising the article. NS is the supervisor of this study and contributed to acquisition and preparation of the data and revising the article. All authors read and approved the final manuscript.

## Pre-publication history

The pre-publication history for this paper can be accessed here:


